# Mycl, activated by Sgk1-phosphorylated Stat3, mediates osteoclastogenesis via *Ctsk* transcriptional regulation

**DOI:** 10.1038/s41598-025-24679-0

**Published:** 2025-11-20

**Authors:** Yiru Wang, Chensong Yang, Fuming Cao, Yuyuan Zhang, Mingzhu Li, Yumei Zhang, Chunxiang Sheng, Li Shao

**Affiliations:** 1 Department of VIP Clinic, Shanghai East Hospital, Tongji University School of Medicine, Shanghai, 200025 China; 2 Department of Traumatic Surgery, Shanghai East Hospital, Tongji University School of Medicine, Shanghai, China; 3 Nanmatou Community Health Service Center, Shanghai, China; 4 Shanghai Acabridge Academy, Shanghai, China; 5 Department of Endocrine and Metabolic Diseases, Shanghai Institute of Endocrine and Metabolic Diseases, Ruijin Hospital, Shanghai Jiao Tong University School of Medicine, Shanghai, 200025 China; 6 Shanghai National Clinical Research Center for Metabolic Diseases, Key Laboratory for Endocrine and Metabolic Diseases of the National Health Commission, Shanghai Key Laboratory for Endocrine Tumor, Ruijin Hospital, Shanghai Jiao Tong University School of Medicine, Shanghai, 200023 China

**Keywords:** Sgk1, Mycl, Stat3 signaling, Bone remodeling, Osteoclastogenesis, Cell biology, Transcription, Metabolic bone disease

## Abstract

Osteoclast differentiation is essential for bone homeostasis, yet its molecular regulation remains incompletely understood. Here, we identify serum/glucocorticoid-regulated kinase 1 (Sgk1), an AGC kinase responsive to hormonal signals, as a key regulator of osteoclastogenesis. Mechanistically, inhibition of Sgk1 reduces Stat3 phosphorylation at Tyr705, leading to the downregulation of Mycl, a less characterized member of the MYC family of transcription factors. We demonstrate that Mycl directly binds to the Ctsk promoter, and functional assays confirm its critical role; Mycl overexpression rescued the osteoclast differentiation impairment caused by Sgk1 inhibition. *In vivo*, treatment with the Sgk1 inhibitor GSK650394 increased trabecular bone mass and enhanced mechanical strength without compromising osteoblast activity. Collectively, our findings define a novel Sgk1-Stat3-Mycl-Ctsk signaling axis that contributes to osteoclastogenesis and suggest that Sgk1 inhibition represent a potential therapeutic strategy for osteoporosis.

**Supplementary Information:**

The online version contains supplementary material available at 10.1038/s41598-025-24679-0.

## Introduction

Bone remodeling, a dynamic process that maintains skeletal integrity, relies on precise coordination between osteoclast-mediated resorption and osteoblast-driven formation^1^. Disruption of this balance, particularly through pathological osteoclast activation, contributes to osteoporosis, inflammatory arthritis, and bone metastatic diseases^2^. The Receptor activator of nuclear factor kappa-B ligand (RANKL)-RANK signaling axis activates nuclear factor of activated T cells 1 (NFATc1), the master transcriptional regulator of osteoclastogenesis that induces expression of bone-resorbing enzymes (e.g., cathepsin K encoded by Ctsk) and cell fusion mediators (e.g., Oc-stamp, Dc-stamp)^3,4^. Beyond the canonical RANKL-RANK-NFATc1 axis, emerging evidence indicates that osteoclast activity integrates diverse systemic signals including metabolic stress and hormonal fluctuations^5–9^. This multilayered regulation suggests the existence of molecular nodes that coordinate extrinsic signals with the core osteoclast differentiation program, though many of these regulatory components remain to be fully characterized.

Among these regulatory components, Sgk1 has been implicated in context-dependent, and at times contrasting roles in osteoclast regulation. Although Sgk1 inhibition suppresses osteoclast differentiation through PI3K-AKT^10^, it has also been reported to promote inflammatory bone erosion through TRAF3/NF-κB in arthritis^11^. This functional duality likely reflects the context-dependent roles of Sgk1 across different physiological and pathological systems. Intriguingly, Sgk1-Stat3 crosstalk drives pathological processes in hepatocytes during ischemia-reperfusion injury via IL-6/Stat3 signaling^12^, in tumor microenvironments via T cell exhaustion mechanisms^13^, and in macrophages via M2 polarization^14^. However, whether the Sgk1-Stat3 interaction regulates Stat3’s pro-osteoclastogenic function—a key driver of osteoporosis and arthritis^15^—remains unknown, leaving a critical gap in targeting bone diseases.

The downstream effectors of Sgk1-Stat3 signaling in osteoclasts remain poorly characterized. While c-MYC is known to drive osteoclastogenesis through NFATc1 activation and metabolic reprogramming^16^, its paralog Mycl, which contains distinct MB0/MB2 domains that enable unique transcriptional regulation^17^, has not yet been examined in bone metabolism. This gap is noteworthy because Stat3 activation has been shown to upregulate Mycl in leukemic T cells^18^, suggesting potential conservation of this regulatory relationship in osteoclasts. Thus, despite the established role of c-MYC, the possibility of a parallel, Stat3-driven regulatory pathway mediated by Mycl in osteoclasts has been overlooked. We therefore hypothesized that Stat3-Mycl signaling may represent a regulatory layer distinct from canonical c-MYC activity, with implications for targeted therapy.

Here, we identify Mycl as a key downstream effector linking Sgk1-Stat3 signaling to osteoclast function. Pharmacological inhibition of Sgk1 with GSK650394 ablates Stat3 phosphorylation at Tyr705, thereby suppressing Mycl expression. Mechanistically, Mycl directly binds the *Ctsk* promoter to activate cathepsin K transcription, revealing a complementary mechanism for osteoclast activation. Our findings identify the Sgk1-Stat3-Mycl-*Ctsk* axis as a potential therapeutic target in pathological bone loss.

## Materials and methods

### Mice

All experimental procedures were approved by the Animal Ethics Committee of Shanghai East Hospital (Approval No. 2025 − 667). The study was conducted in accordance with the Guide for the Care and Use of Laboratory Animals and reported following ARRIVE guidelines. Male C57BL/6 mice (16 weeks old) were obtained from Jiangsu HuaChuang XinNuo Medical Technology Co., Ltd. (Jiangsu, China). The mice were randomly divided into two groups: the control (Con) group and the GSK650394 (GSK) -treated group. Mice in the Con group received vehicle injections, while those in the GSK group were administered GSK (30 mg/kg) intraperitoneally (i.p.) dissolved in the vehicle. Injections were administered every other day for 8 weeks. The vehicle solution consisted of 10% dimethyl sulfoxide, 40% polyethylene glycol 300, 5% Tween-80, and 45% saline.

The mice were housed under specific pathogen-free (SPF) conditions with 12-hour light/dark cycles at 22 °C. They had ad libitum access to standard rodent chow and water throughout the experimental period. At the end of the 8-week treatment period, all mice were humanely euthanized under deep isoflurane anesthesia (4%) followed by controlled CO₂ asphyxiation, and bone tissues were collected for subsequent analyses. All procedures were performed in accordance with the approved guidelines.

### Cell culture

Primary bone marrow-derived macrophages (BMMs) and RAW264.7 murine macrophage cell line (obtained from Chinese Academy of Sciences, Shanghai) were used. RAW264.7 cells were cultured in α-MEM (Thermo Fisher Scientific, USA) supplemented with 10% FBS (Thermo Fisher Scientific) and 1% penicillin-streptomycin (P/S, Thermo Fisher Scientific) at 37 °C with 5% CO₂, passaged every 2–3 days at a 1:5 ratio.

For BMMs isolation, male C57BL/6 mice (8-week-old) were obtained from Vital River Laboratory Animal Technology Co., Ltd. (Shanghai, China) and euthanized in accordance with institutional ethical guidelines. Bone marrow cells were aseptically harvested from femurs and tibias, rinsed with α-MEM, and seeded in α-MEM containing 10% FBS and 1% P/S. After 24 h of incubation, nonadherent cells were collected and differentiated in fresh α-MEM medium supplemented with 10% FBS, 1% P/S, and 30 ng/mL macrophage colony-stimulating factor (M-CSF; BioLegend, California, USA). Adherent cells were maintained under these conditions until reaching 90% confluence.

### Bone sample preparation and micro-computed tomography (micro-CT) analysis

The right femurs were dissected and fixed in 4% paraformaldehyde at 4 °C for 48 h, then stored in 70% ethanol. Bone microarchitecture was analyzed using a SkyScan 1176 micro-CT system (Bruker, Kontich, Belgium) with 8 μm isotropic resolution.

For trabecular bone analysis, we examined 224 consecutive slices adjacent to the distal growth plate. Cortical bone was assessed across 66 slices (450–516) above the growth plate. Microstructural parameters were quantified using CTAn software (Bruker) in accordance with the established guidelines of the American Society for Bone and Mineral Research (ASBMR)^19^. Three-dimensional (3D) reconstructions were generated using CTvox software (Bruker) for morphological visualization.

### Three-point bending test for biomechanical analysis

The left femurs were dissected and tested using an Instron 5569 testing machine (Instron, Inc., Norwood, USA). Femurs were placed on supports 6 mm apart and loaded at the midpoint at 1 mm/min until fracture. Load-displacement data were recorded at 100 Hz.

Force-deflection curves were generated using MATLAB (MathWorks Inc., Natick, MA, USA) to calculate maximum load (ultimate strength), elastic modulus (stiffness), energy to failure (toughness), and yield load for mechanical property assessment.

### Bone histomorphometry

Femurs were dissected, fixed in 4% PFA (24 h, 4 °C), and decalcified in 23% ethylenediaminetetraacetic acid (EDTA; 3 days, 4 °C). Paraffin-embedded samples were sectioned at 5 μm thickness. Tartrate-resistant acid phosphatase (TRAP) staining was performed using a commercial kit (387 A-1KT, Sigma-Aldrich, St. Louis, MO, USA) to identify osteoclasts (TRAP-positive multinucleated cells). Histomorphometric analysis was performed in accordance with the standard nomenclature and guidelines established by the ASBMR^20^. ImageJ software was used to quantify the osteoclast number per bone trabecular parameter (N.Oc/B.Pm) and the osteoclast surface relative to the bone trabecular surface (Oc.S/BS). Measurements were conducted in trabecular bone regions adjacent to the growth plate.

### Measurement of serum biomarkers

After 6-hour fasting, blood samples were collected and clotted at room temperature for 1 h. Serum was separated by centrifugation (3000 rpm, 10 min) and stored at -80 °C. Bone turnover markers were measured using ELISA kits: N-terminal propeptide of type I procollagen (PINP; USCN Life Science, Wuhan, China) for bone formation and C-terminal telopeptide of type I collagen (CTX-I; Immunodiagnostic Systems, Tyne & Wear, UK) for bone resorption, following manufacturer protocols.

### *In vitro* osteoclast differentiation assay

Osteoclast differentiation was induced in BMMs by culturing cells in 96-well plates in α-MEM medium supplemented with 10% FBS, 1% P/S, 50 ng/mL RANKL (BioLegend), and 30 ng/mL M-CSF for 4 days. At the onset of differentiation, BMMs were treated with 1 µM GSK. Following the differentiation period, osteoclast formation was evaluated using a TRAP staining kit according to the manufacturer’s protocol. Osteoclasts were defined as TRAP-positive multinucleated cells containing three or more nuclei. Cells were imaged using an Olympus microscope, and the number of TRAP-positive multinucleated cells (≥ 3 nuclei) was quantified and normalized to the well area to yield osteoclasts per square centimeter (OCs/cm²).

### Adenoviruses Preparation and transduction

BMMs and RAW264.7 cells were seeded onto cell culture plates and allowed to adhere for 24 h. Cells were then transduced with adenoviral constructs at a multiplicity of infection (MOI) of 100 for 6 h, followed by replacement with complete medium. Experiments were conducted 24 h post-transduction.

For Ad-*Sgk1* construction, the full-length murine Sgk1 coding sequence was amplified from a cDNA library (Genechem, Shanghai, China) using the following primers: *Sgk1*: 5′-AGGTCGACTCTAGAGGATCCCGCCACCATGACCGTCAAAGCCGAGGCTGCTC-3′ (forward); *Sgk1*: 5′-TCCTTGTAGTCCATACCGGTGAGGAAGAATCCACAGGAGGTGCATAG-3′ (reverse).

For Ad-*Mycl* construction, the full-length murine *Mycl* coding sequence was amplified using the following primers: *Mycl*: 5′-AGGTCGACTCTAGAGGATCCCGCCACCATGGACTTCGACTCGTATCAG-3′ (forward); *Mycl*: 5′-TCCTTGTAGTCCATACCGGTGTAGCCACTGAGGTACGCGATTC-3′ (reverse).

The adenovirus vector plasmid GV314 (CMV-MCS-3FLAG-SV40-EGFP) was purchased from Genechem Biotechnology Co., Ltd. The vector and *Sgk1*/*Mycl* gene sequences were digested with AgeI and BamHI and ligated using the In-fusion recombination method. Recombinant vectors were verified by DNA sequencing.

Ad-sh*Sgk1* encoded a short hairpin RNA (shRNA) targeting the *Sgk1* transcript (5′-GCGGAATGTTCTGTTGAAGAA-3′). A control adenovirus expressing scrambled shRNA (sh-Ctrl, 5′-TTCTCCGAACGTGTCACGT-3′) was also constructed by Genechem Biotechnology Co., Ltd.

### qRT-PCR

Total RNA was extracted using TRIzol reagent, and complementary DNA (cDNA) was synthesized from the isolated RNA using the HiScript III RT SuperMix for qPCR (+ gDNA wiper) kit (Vazyme, Nanjing, China). qRT-PCR was performed on the Applied Biosystems 7300 Real-Time PCR System (Foster City, CA, USA) with ChamQ Universal SYBR qPCR Master Mix (Vazyme). The relative mRNA expression levels of target genes were normalized to the housekeeping gene *18S* using the 2^^(−ΔΔCT)^ method. The primer sequences used for qRT-PCR were as follows (mouse genes): *18S*, 5′- GCAATTATTCCCCATGAACG-3’ (forward), 5′- GGCCTCACTAAACCATCCAA − 3′ (reverse); *Mycl*: 5′- CGAGAACGGCTGGAGAGAGT-3’ (forward), 5′- CCACGGTCACCACGTCAATC − 3′(reverse); *Nfatc1*: 5′- TGGGAGATGGAAGCAAAGACTGA-3’ (forward), 5′-CATTGGCAGGAAGGTACGTGAA − 3′ (reverse); *Ctsk*: 5′- CGCTCACAGTAGCCACGCTT-3’ (forward), 5′- CCGAGAGATTTCATCCACCTTGCT − 3′ (reverse); *Acp5*: 5′- CACTCCCACCCTGAGATTTGT-3’ (forward), 5′- CATCGTCTGCACGGTTCTG − 3′ (reverse); *Oc-stamp*: 5′- CTGTAACGAACTACTGACCCAGC − 3’ (forward), 5′- CCCAGGCTTAGGAAGACGAAG − 3′ (reverse); *Dc-stamp*: 5′- GGGGACTTATGTGTTTCCACG − 3’ (forward), 5′- ACAAAGCAACAGACTCCCAAAT − 3′ (reverse); *Jak2*: 5′- GGATGTGAGTGCGGGCCAAG − 3’ (forward), 5′- GTAAGGCAGGCCATTCCCATC − 3′ (reverse); *c-Myc*: 5′- GAGAAGAGGCAAACCCCTGC − 3’ (forward), 5′- GAGCTTGTGCTCGTCTGCTT − 3′ (reverse); *Mycn*: 5′- CTGCGGTCACTAGTGTGTCTG − 3’ (forward), 5′- ATCGTGAAAGTGGTTACCGCC − 3′ (reverse).

### Western blot

Total protein was extracted using RIPA buffer (Beyotime Biotechnology) supplemented with a phosphatase inhibitor cocktail, protease inhibitor cocktail (both from MedChemExpress), and phenylmethylsulfonyl fluoride (PMSF; Beyotime Biotechnology). Histone proteins were isolated using the EpiQuik Total Histone Extraction Kit (Epigentek, New York, NY, USA), while cytosolic and nuclear proteins were fractionated using the Nuclear and Cytoplasmic Protein Extraction Kit (CWBIO, Shanghai, China). Protein concentrations were quantified using the BCA Protein Assay Kit (Thermo Fisher Scientific).

For Western blot analysis, proteins were separated by SDS-PAGE using precast gels (EpiZyme Biotechnology Co., Ltd, Shanghai, China) and transferred onto polyvinylidene difluoride (PVDF) membranes (Millipore, Burlington, MA, USA). Membranes were blocked with 5% bovine serum albumin (BSA; Sigma, St. Louis, MO, USA) for 1 h at room temperature. Subsequently, membranes were incubated with primary antibodies at 4 °C overnight, followed by incubation with horseradish peroxidase (HRP)-conjugated secondary antibodies for 1 h at room temperature. After washing, protein bands were visualized using enhanced chemiluminescence (ECL) substrate (Meilunbio, China) and imaged using the Touch Imager XLi system (e-BLOT, Shanghai, China). The following antibodies were used: NFATc1 (Santa Cruz, #sc-7294, 1:500), β-Actin (Santa Cruz, #sc-47778, 1:2000), HSP90 (Santa Cruz, #sc-13119, 1:1000), Sgk1 (Abcam, # ab43606, 1:500), FLAG (Proteintech, #20543-1-AP, 1:2000), Histone H3 (CST, #4499, 1:2000), GAPDH (CST, #2118, 1:5000), α-Tubulin (CST, #2148, 1:2000), p-Stat3 (Tyr705) (CST, #9145, 1:1000), Stat3 (CST, #9139, 1:1000), p-Akt (Ser473) (CST, # 9271, 1:1000), Akt (CST, # 9272, 1:1000), Jak2 (CST, # 3230, 1:1000), p-Src (Tyr416) (CST, # 2101, 1:1000), Src (CST, # 2108, 1:1000), Mycl (Absin, #abs152608, 1:500).

### Luciferase reporter assay

Luciferase reporter assays were performed to investigate the regulatory effects of GSK on *Mycl* and the role of Mycl in *Ctsk* regulation.

For the GSK-*Mycl* regulation study, RAW264.7 cells were seeded in 24-well plates at a density of 1 × 10^^5^ cells per well and cultured for 24 h. Cells were transiently co-transfected with a mixture of the Renilla luciferase plasmid (internal control) and the Mycl luciferase reporter construct (Genechem) using Lipofectamine 3000 reagent (Invitrogen, Carlsbad, CA, USA). After 24 h of transfection, cells were treated with GSK for an additional 6 h. The *Mycl* promoter region was cloned into the GV534 vector upstream of the firefly luciferase gene. The primer sequences used for amplification were as follows:

5′-CGGTACCTGAGCTCGCTAGCGACTGCTCTTCCAAAGGTCC-3 (forward), 5′-AGTACCGGATTGCCAAGCTTGCTCCCGGGATCGACGCGGGAGC-3′ (reverse).

For the Mycl-*Ctsk* regulation study, RAW264.7 cells were co-transfected with the Renilla luciferase plasmid and the *Ctsk* luciferase reporter construct (Genechem) for 8 h, followed by transfection with the *Mycl* overexpression adenovirus (Ad-*Mycl*) for 36 h. The *Ctsk* promoter region was cloned into the GV534 vector upstream of the firefly luciferase gene. The primer sequences used for amplification were as follows:

5′- CGGTACCTGAGCTCGCTAGCTTTTAAAACAAAGTATTTTTTC − 3′ (forward), 5′- AGTACCGGATTGCCAAGCTTGCGGAAGTCAACTCCAGCAGTGG − 3′ (reverse). After treatment, cells were lysed using passive lysis buffer from the Firefly & Renilla Luciferase Reporter Assay Kit (Meilunbio, China). Luciferase activity was measured using the Dual-Glo Luciferase Assay System (Promega, Madison, WI, USA). Firefly luciferase activity was normalized to Renilla luciferase activity to account for variations in transfection efficiency.

### RNA-sequencing analysis

Total RNA was extracted from BMMs cultured under the following conditions: (1) Basal group (M-CSF alone), (2) RANKL-induced group (M-CSF + RANKL), and (3) GSK-treated group (M-CSF + RANKL + GSK). After 4 days of incubation, RNA was isolated using TRIzol reagent. RNA sequencing and subsequent bioinformatics analyses were performed by Romics Biotechnology Co., Ltd. (Shanghai, China). Sequencing libraries were prepared using the NEBNext UltraTM RNA Library Prep Kit (NEB, Cat# E7490), and purification was conducted using AMPure XP beads. High-throughput sequencing was performed on the Illumina NovaSeq 6000 platform with paired-end 150 bp (PE150) reads.

Differentially expressed genes (DEGs) between the groups were identified based on the following criteria: |log2FC| ≥1, adjusted *p*-value (*p.* adj) < 0.05. Functional enrichment analysis of the differentially expressed genes was performed using the clusterProfiler R package, focusing on Gene Ontology (GO) biological processes and Kyoto Encyclopedia of Genes and Genomes (KEGG) pathways. Significantly enriched terms and pathways were identified using a hypergeometric test with a Benjamini-Hochberg false discovery rate (FDR) adjustment, and those with a *p.* adj < 0.05 were considered statistically significant.

### Chromatin Immunoprecipitation (ChIP)

For the analysis of Mycl occupancy on the *Ctsk* promoter, BMMs were transduced with either a control vector or FLAG-tagged *Mycl* overexpression adenovirus for 24 h, followed by culture in osteoclastogenic medium for 4 days. The ChIP assay was performed using the ChIP Assay Kit (17–295, Merck, Darmstadt, Germany). Briefly, BMMs were cross-linked with 1% formaldehyde, and the reaction was quenched with glycine. Chromatin was fragmented into DNA segments ranging from 200 to 1000 bp using sonication. Immunoprecipitation was conducted using antibodies specific to FLAG or IgG (as a negative control). The precipitated DNA was analyzed by qPCR using primers targeting the promoter regions of *Ctsk*. The primer sequences were as follows: *Ctsk* promoter: 5′-ATGTTGAGGGGACAGAGGTG-3′ (forward); 5′-TGGCTGTACATCAAGGGAGG-3′ (reverse).

### Statistical analysis

All quantitative data are presented as mean ± standard deviation (SD). Statistical significance was evaluated by unpaired Student’s t test with a two-tailed distribution for two groups or ANOVA for multiple groups. Differences were considered to be statistically significant when *p* < 0.05. All analyses were performed using GraphPad Prism software, version 8.0.2.

## Results

### GSK treatment improves bone quality and suppresses osteoclast activity

**Fig. 1:**
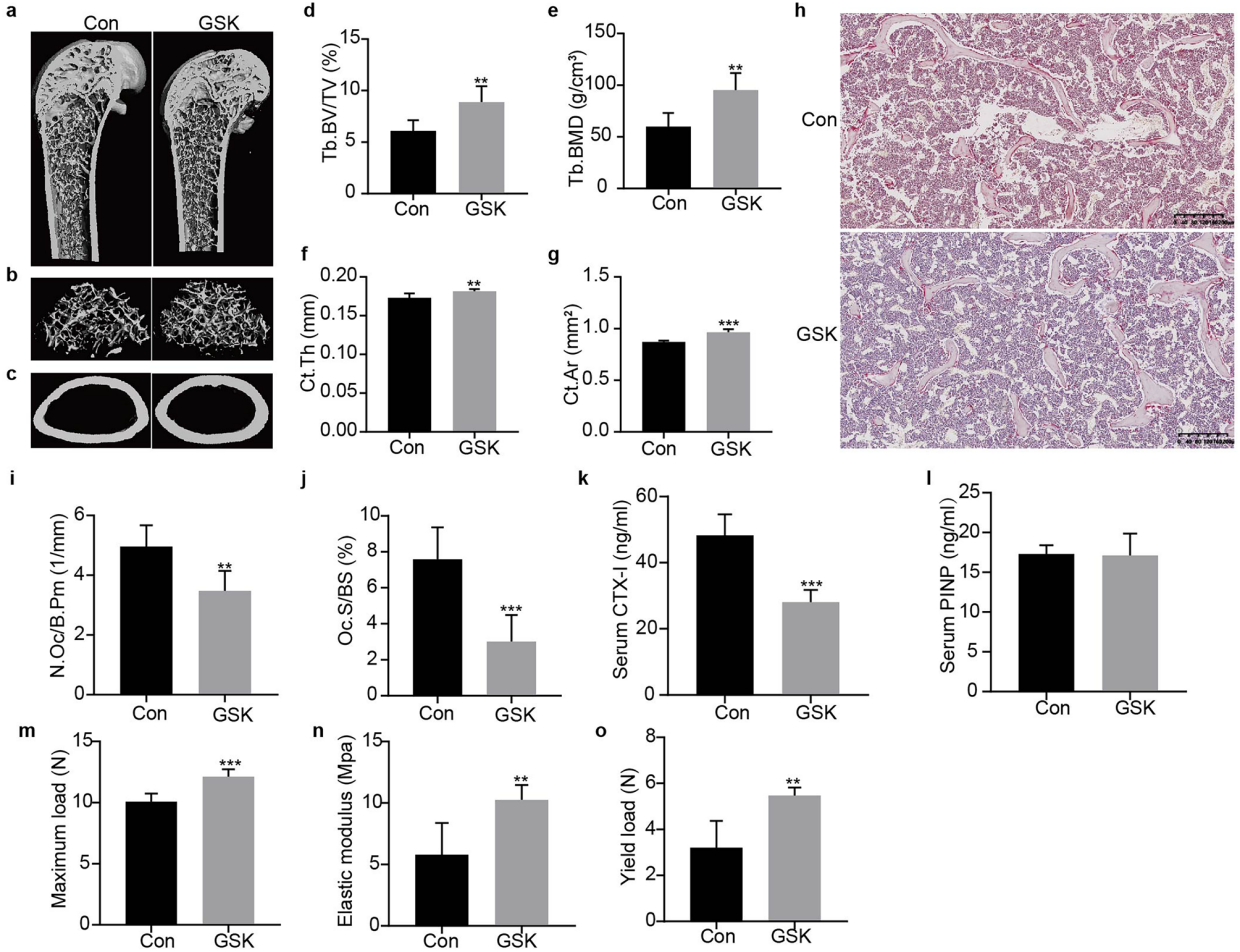
Effects of GSK treatment on bone metabolism and microstructure in mice. (**a**–**c**) Micro-CT analysis showing 3D reconstructed images of: coronal sections, trabecular bone, and cortical bone in distal femurs. (**d**–**g**) Quantitative micro-CT analysis of: BV/TV, BMD, Ct.Th, and Ct.Ar. (**h**) Representative images of TRAP staining in distal femoral epiphyses. (**i**, **j**) The quantification of N.Oc/B.Pm and Oc.S/BS. (**k**, **l**) Serum levels of bone turnover markers were measured by ELISA: CTX-I as a bone resorption marker; PINP as a bone formation marker. (**m**–**o**) Biomechanical properties of femurs assessed by three-point bending test: maximum load, elastic modulus, and yield strength. Data are presented as mean ± SD (*n* = 6 per group). ^**^p < 0.01, ^***^p < 0.001 versus control (Con) group.

To investigate the regulatory role of Sgk1 in bone metabolism, we administered the Sgk1 inhibitor GSK (30 mg/kg, i.p.) to 16-week-old male C57BL/6 mice every other day for 8 weeks. Micro-CT analysis (Fig. 1a-c) revealed that GSK-treated mice exhibited significant improvements in bone microstructure compared to vehicle controls, including increased trabecular bone volume/total volume (BV/TV, *p*<0.01; Fig. 1d), elevated bone mineral density (BMD, *p*<0.01; Fig. 1e), enhanced cortical thickness (Ct.Th, *p*<0.01; Fig. 1f), and expanded cortical area (Ct.Ar, *p*<0.001; Fig. 1g).

Consistent with suppressed bone resorption, TRAP staining of distal femurs showed a marked reduction in TRAP-positive osteoclasts in treated animals (Fig. 1h). Histomorphometric analysis confirmed attenuated osteoclast activity, with significant decreases in osteoclast number (N.Oc/B.Pm, *p*<0.01; Fig. 1i) and surface coverage (Oc.S/BS, *p*<0.001; Fig. 1j). Serum biomarker analysis further supported these findings, showing significant reductions in the bone resorption marker CTX-I (*p*<0.001; Fig. 1k) while levels of the bone formation marker PINP remained unchanged (Fig. 1l).

These microarchitectural improvements were accompanied by enhanced biomechanical properties. Three-point bending tests demonstrated increased maximum load capacity (*p*<0.001; Fig. 1m), elastic modulus (*p*<0.01; Fig. 1n), and yield strength (*p*<0.01; Fig. 1o) in treated femurs. Collectively, these data indicate that Sgk1 inhibition suppresses osteoclast activity and improves bone quality without detectable adverse effects on osteoblast activity.

### Sgk1 Inhibition attenuates osteoclast differentiation and marker expression in BMMs

**Fig. 2:**
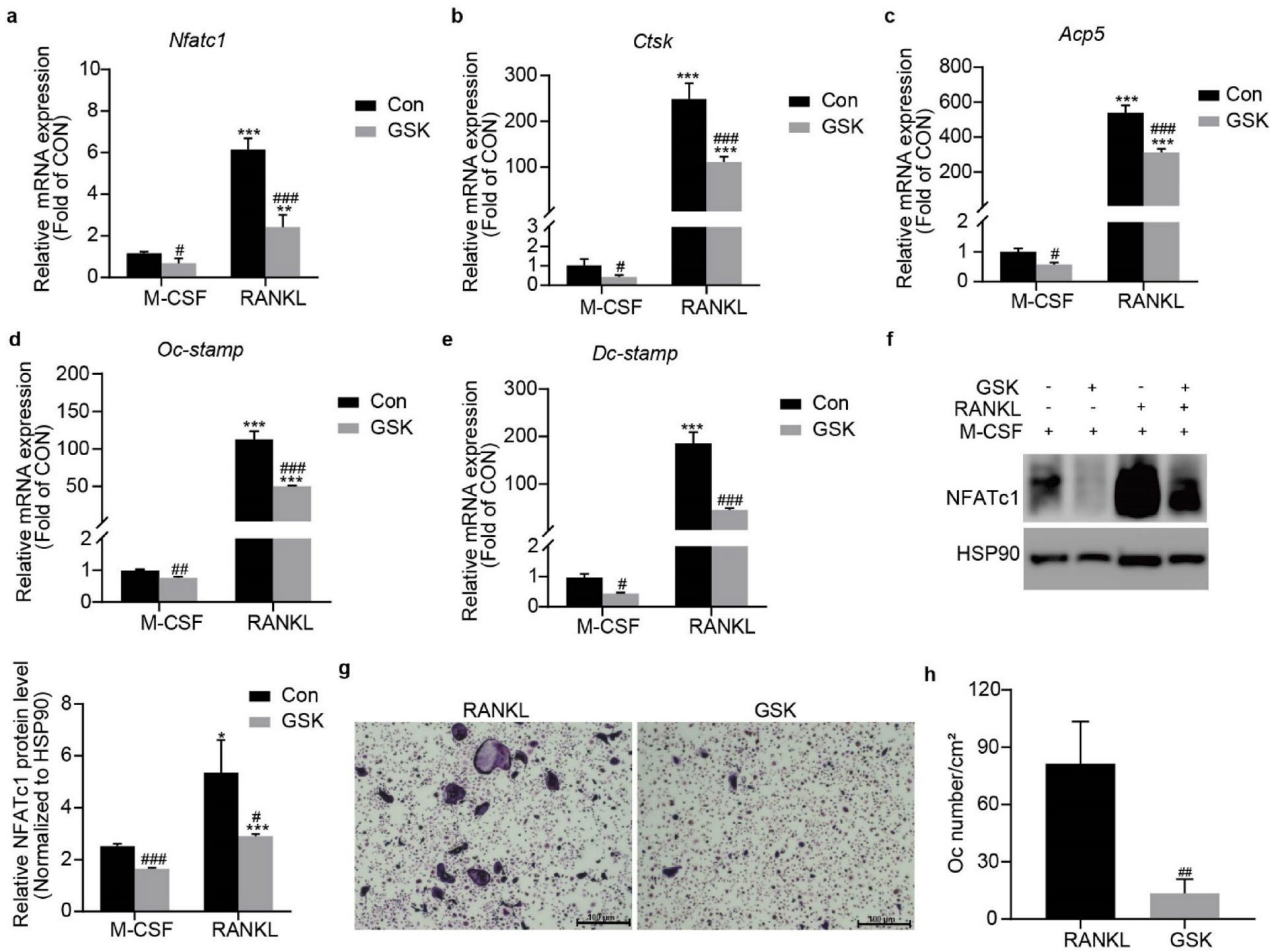
Effects of GSK on osteoclast differentiation and marker expression in BMMs. BMMs were treated with or without GSK under: Differentiation conditions (M-CSF + RANKL); Basal conditions (M-CSF alone) for 4 days. (**a**–**e**) *Nfatc1*, *Ctsk*, *Acp5*, *Oc-stamp*, *Dc-stamp* mRNA expression by qRT-PCR. (**f**) NFATc1 protein expression was determined by Western blot. The blot image shows adjacent lanes from the same membrane. Band intensities were quantified with ImageJ software and normalized to HSP90. Original blots are presented in Supplementary Fig. 5. (**g**) Representative TRAP staining images (differentiation conditions). (**h**) Quantification of osteoclasts (OCs/cm²) from cultures in 96-well plates. Data represent mean ± SD from three independent experiments. ^*^p < 0.05,  ^**^p < 0.01, ^***^p < 0.001 vs. M-CSF; ^#^*p* < 0.05, ^##^*p* < 0.01, ^###^*p* < 0.001 vs. Con group.

Having established the osteoclast-suppressing effects of Sgk1 inhibition* in* *vivo*, we next sought to delineate the underlying molecular mechanisms using BMMs. BMMs were treated with the Sgk1 inhibitor GSK (1 µM) or vehicle control under basal (M-CSF alone) or differentiation (M-CSF + RANKL) conditions for 4 days. qRT-PCR analysis revealed that GSK significantly suppressed the expression of osteoclast-specific genes (*Nfatc1*, *Ctsk*, *Acp5*, *Oc-stamp*, and *Dc-stamp*) under both conditions (Fig. 2a-e). Western blot analysis confirmed decreased NFATc1 protein levels in GSK-treated cells (Fig. 2f). TRAP staining demonstrated markedly fewer TRAP-positive multinucleated cells (≥ 3 nuclei) in treated groups (Fig. 2g, h).

**Fig. 3:**
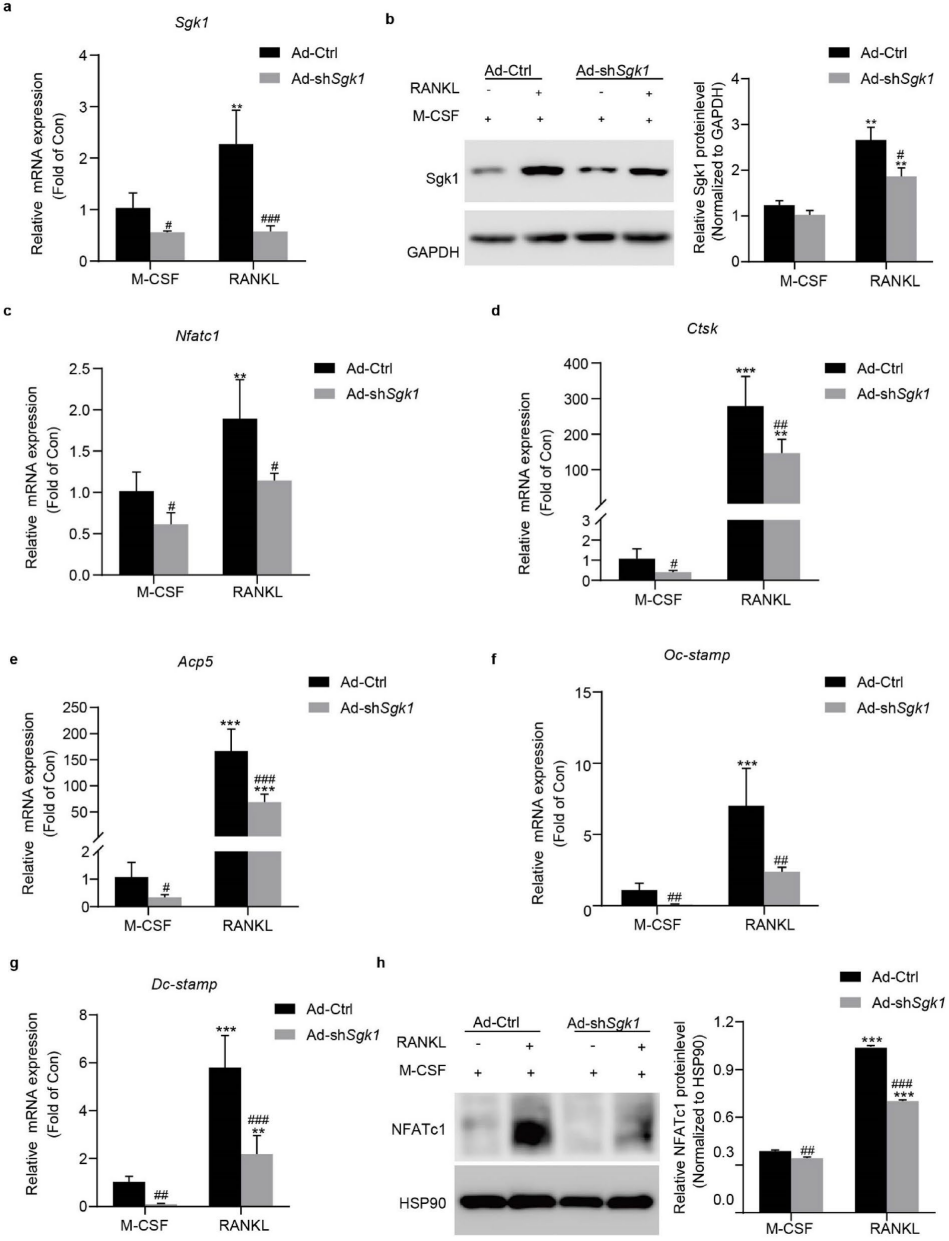
Effects of *Sgk1* knockdown on osteoclastogenic marker expression in BMMs. BMMs transfected with Ad-Ctrl or Ad-shSGK1 adenovirus and cultured under differentiation (M-CSF + RANKL) or basal (M-CSF alone) conditions for 4 days. (**a**) *Sgk1* mRNA expression by qRT-PCR. (**b**) Sgk1 protein levels were analyzed by Western blot. The blot image shows adjacent lanes from the same membrane. Band intensities were quantified with ImageJ software and normalized to GAPDH. Original blots are provided in Supplementary Fig. 6. (**c**–**g**) *Nfatc1*, *Ctsk*, *Acp5*, *Oc-stamp*, *Dc-stamp* mRNA expression by qRT-PCR. (**h**) NFATc1 protein levels were analyzed by Western blot. The blot image shows adjacent lanes from the same membrane. Band intensities were quantified with ImageJ software and normalized to HSP90. Original blots are provided in Supplementary Fig. 7. Data are presented as mean ± SD from three independent experiments. ^**^p < 0.01, ^***^p < 0.001 vs. M-CSF; ^#^*p* < 0.05, ^##^*p* < 0.01, ^###^*p* < 0.001 vs. Ad-Ctrl group.

To further define Sgk1’s role in osteoclastogenesis, we performed genetic knockdown using Ad-sh*Sgk1*. Efficient Sgk1 knockdown was confirmed by qRT-PCR and Western blot (Fig. 3a, b). Similar to pharmacological inhibition, Ad-sh*Sgk1* transduction downregulated osteoclast-related genes (*Nfatc1*, *Ctsk*, *Acp5*, *Oc-stamp*, and *Dc-stamp*) regardless of RANKL stimulation (Fig. 3c-g), with parallel reduction in NFATc1 protein (Fig. 3h). These findings indicate that Sgk1 contributes to osteoclast differentiation both under basal and RANKL-stimulated conditions.

### GSK inhibits osteoclast differentiation via *Mycl* transcriptional suppression and Stat3 deactivation

**Fig. 4:**
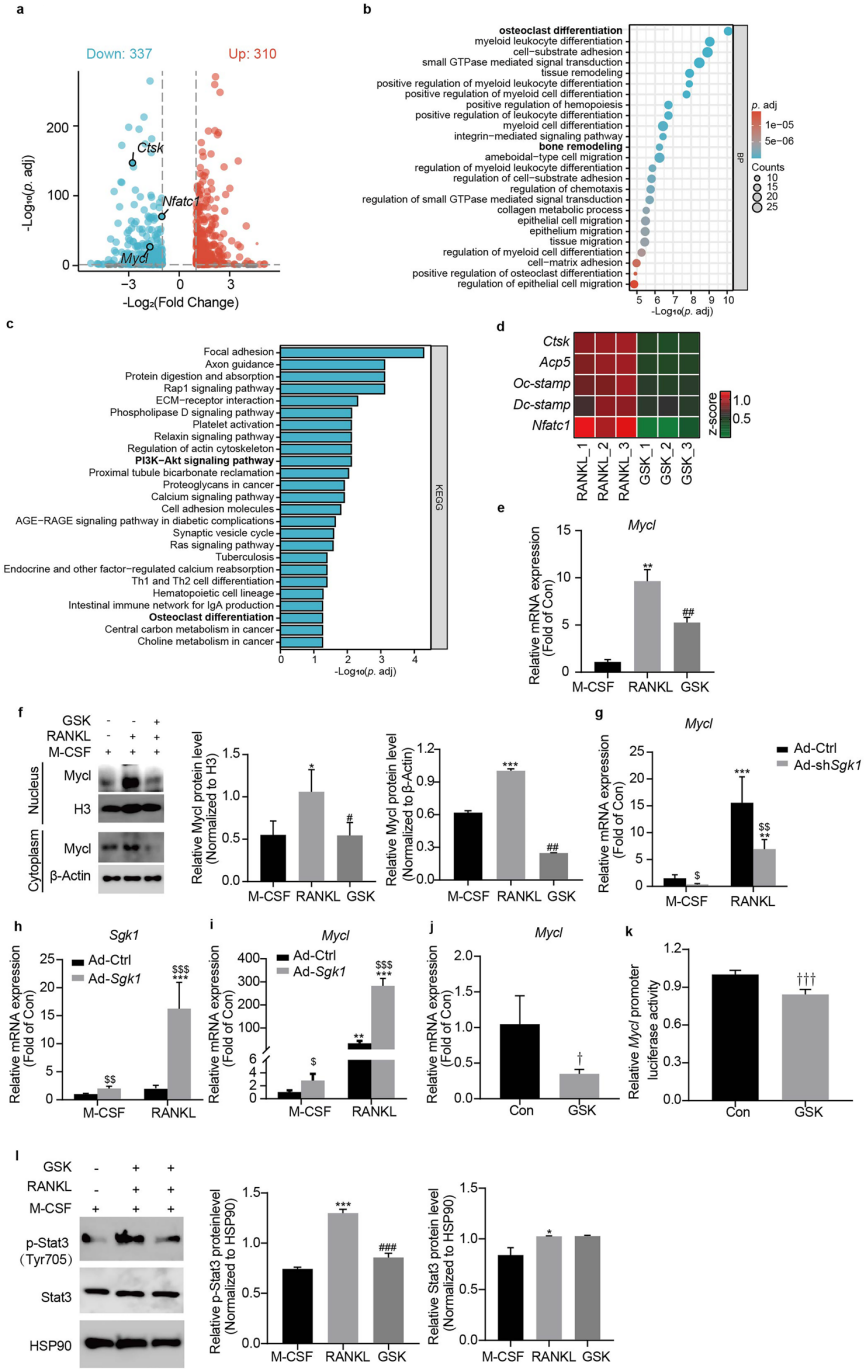
GSK inhibits osteoclastogenesis by downregulating *Mycl* and p-Stat3. (**a**) Volcano plot showing differentially expressed genes (|log2FC| ≥1, *p.* adj < 0.05) in GSK-treated versus untreated BMMs cultured in differentiation conditions (M-CSF + RANKL) for 4 days. (**b**) GO enrichment analysis of the top 25 biological processes associated with 337 GSK-downregulated genes. Processes ranked by -log10(*p.* adj). (**c**) KEGG pathway enrichment analysis of the top 25 pathways associated with 337 GSK-downregulated genes. Pathways ranked by -log10(*p.* adj). (**d**) Heatmap visualization of normalized expression levels for osteoclast-specific genes (*Nfatc1*, *Ctsk*, *Acp5*, *Oc-stamp*, *Dc-stamp*) in GSK-treated versus control BMMs under differentiation conditions. (**e**) *Mycl* mRNA expression levels by qRT-PCR in BMMs cultured under basal, differentiation, or differentiation with GSK treatment conditions for 4 days. (**f**) Mycl protein expression in cytoplasmic and nuclear fractions was determined by Western blot in BMMs cultured under basal, differentiation, or GSK-treated differentiation conditions for 4 days. The blot image shows adjacent lanes from the same membrane. Band intensities were quantified with ImageJ and normalized to H3 (nuclear) and β-Actin (cytoplasmic). Original blots are provided in Supplementary Fig. 8. (**g**) *Mycl* mRNA levels by qRT-PCR in BMMs transfected with Ad-sh*Sgk1* or Ad-Ctrl and cultured in differentiation or basal conditions for 4 days. (**h**, **i**) *Sgk1* and *Mycl* mRNA expression by qRT-PCR in BMMs transfected with Ad-*Sgk1* or Ad-Ctrl and cultured in differentiation or basal conditions for 48 h. (**j**) *Mycl* mRNA expression in RAW264.7 cells treated with 1 µM GSK for 6 h. (**k**) *Mycl* promoter activity in RAW264.7 cells treated with vehicle or 1 µM GSK for 6 h (luciferase reporter assay). (**l**) Stat3 and p-Stat3 (Tyr 705) protein levels were determined by Western blot in BMMs under non-induced (M-CSF), RANKL-induced, or RANKL + GSK conditions. The blot image shows adjacent lanes from the same membrane. Band intensities were quantified with ImageJ and normalized to HSP90. Original blots are provided in Supplementary Fig. 9. Data are expressed as means ± SD from three independent experiments. ^*^p < 0.05, ^**^p < 0.01, ^***^p < 0.001 vs. M-CSF; ^#^*p* < 0.05, ^##^*p* < 0.01, ^###^*p* < 0.001  vs. RANKL group; ^$^*p* < 0.05, ^$$^*p* < 0.01, ^$$$^*p* < 0.001 vs. Ad-Ctrl; ^†^ *p* < 0.05, ^†††^ *p* < 0.001 vs. Con group.

To explore the molecular mechanisms underlying GSK-mediated inhibition of osteoclast differentiation, we performed transcriptomic profiling of RANKL-stimulated BMMs treated with or without GSK. RNA-seq analysis identified 647 DEGs (|log2FC| ≥ 1, *p.* adj<0.05), with 52% (337/647) downregulated in GSK-treated BMMs (Fig. 4a). Downregulated DEGs were enriched in osteoclast differentiation and bone remodeling pathways (Fig. 4b). KEGG pathway analysis revealed broad suppression of multiple signaling pathways (Fig. 4c). The most significantly enriched was focal adhesion, which is essential for osteoclast adhesion and migration. Critically, pathways directly governing osteoclast differentiation - including the osteoclast differentiation pathway itself (mmu04380) - were also strongly suppressed. As the PI3K-AKT pathway (mmu04151) is a well-established critical regulator of osteoclast differentiation, and was also identified in our KEGG analysis, we hypothesized it to be a key mechanistic target of GSK. This was confirmed functionally, as both GSK treatment and Sgk1 knockdown significantly reduced Akt phosphorylation (Supplementary Fig. 1a, b). Consistent with these findings, key osteoclast genes (*Nfatc1*, *Ctsk*, *Acp5*, *Oc-stamp*, *Dc-stamp*) showed pronounced downregulation (Fig. 4d).

To identify candidate mediators of GSK’s effect, we focused on genes that were most strongly induced by RANKL and whose expression was substantially suppressed by GSK. *Mycl* was among the top downregulated candidates (Supplementary Fig. 2), making it a compelling candidate for further investigation. While c-MYC promotes osteoclastogenesis and MYCN may regulate bone homeostasis via N-myc downstream regulated gene 1 (NDRG1)^21,22^, Mycl’s role in osteoclast biology remained undefined. This downregulation was confirmed at both mRNA (Fig. 4e) and protein levels (Fig. 4f). Genetic manipulation further validated this relationship - Sgk1 knockdown decreased (Fig. 4g), while Sgk1 overexpression (Fig. 4h) increased *Mycl* expression (Fig. 4i). Crucially, these manipulations in BMMs under basal conditions (without RANKL stimulation) demonstrated that Sgk1 intrinsically regulates Mycl independent of differentiation signals.

Mechanistically, to directly probe this cell-autonomous regulatory relationship isolated from the complexity of RANKL-induced differentiation, we treated RAW264.7 cells with GSK (1 µM, 6 h) in the absence of RANKL. This treatment reduced both *Mycl* mRNA levels and promoter activity (Fig. 4j, k). This observation in a simplified system is consistent with the regulation observed in primary BMMs under basal conditions (Fig. 4g, i), suggesting the presence of an Sgk1-Mycl regulatory relationship in osteoclast precursors. Since Stat3 Tyr705 phosphorylation regulates *Mycl* in hematopoietic cells^18^, we examined this relationship in osteoclasts. GSK specifically decreased p-Stat3 (Tyr705) without affecting total Stat3 in BMMs (Fig. 4l). We next investigated the upstream mechanism and found that GSK treatment or Sgk1 knockdown markedly reduced the protein (Supplementary Fig. 3a, b), but not mRNA (Supplementary Fig. 3c, d), levels of Jak2, a major kinase responsible for Stat3 tyrosine phosphorylation^23^, without affecting Src protein levels or phosphorylation (Supplementary Fig. 3e). This inhibition of Stat3 activation is consistent with the reduced expression of both the novel target Mycl and the key transcriptional regulator Nfatc1 ^15^ (as shown in Figs. 2a and f and 3c and h).

### Mycl promotes osteoclast differentiation and directly regulates *Ctsk* transcription

**Fig. 5:**
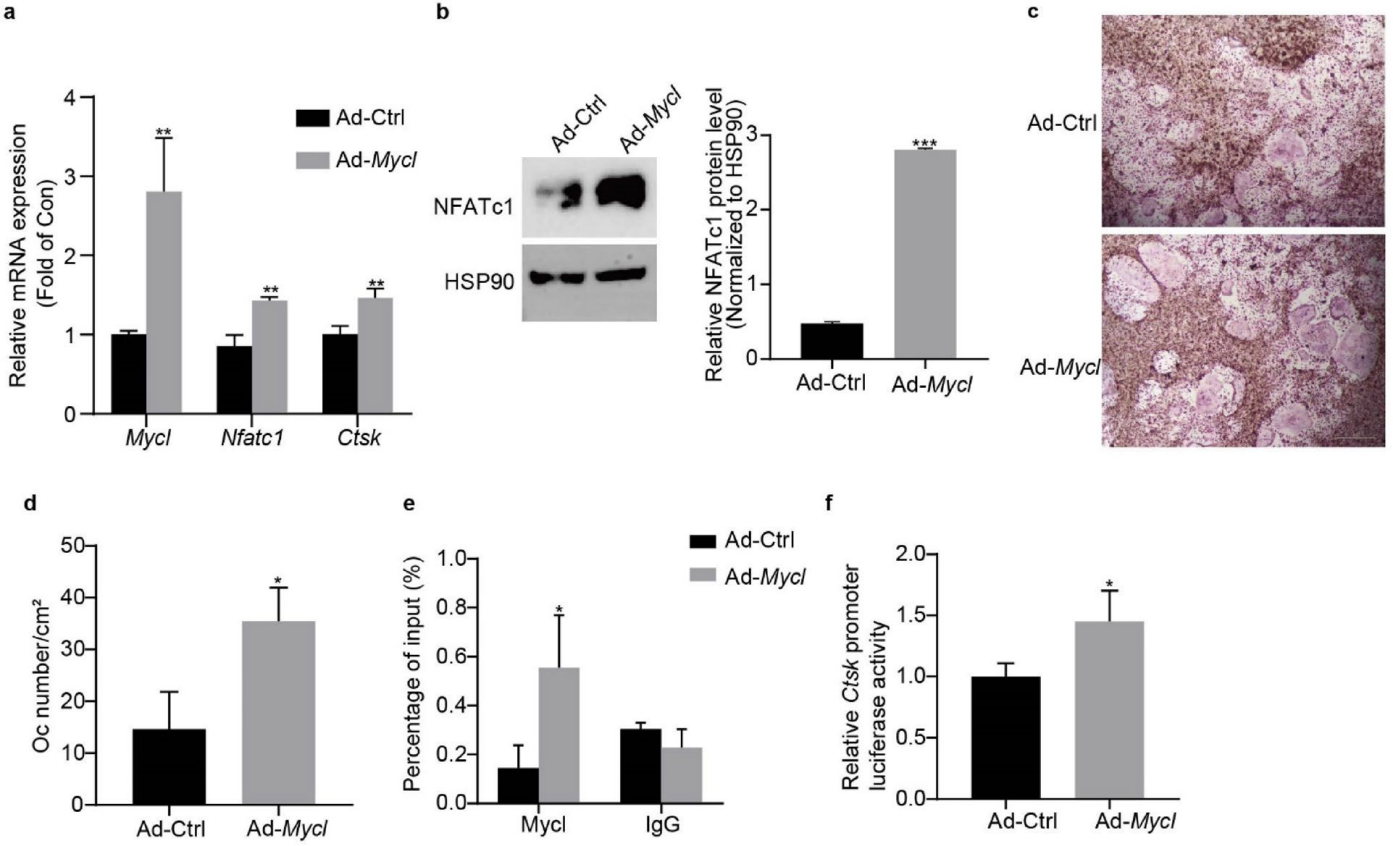
Mycl promotes osteoclast differentiation and directly regulates *Ctsk* transcription. (**a**) mRNA levels of *Mycl* and osteoclast markers (*Nfatc1*, *Ctsk*) in BMMs transfected with Ad-Ctrl or Ad-*Myc*l after 4 days of differentiation. (**b**) NFATc1 protein levels in Ad-Ctrl- or Ad-*Mycl*-transfected BMMs were analyzed by Western blot after 4 days of differentiation. The blot image shows adjacent lanes from the same membrane. Band intensities were quantified with ImageJ and normalized to HSP90. Original blots are provided in Supplementary Fig. 10. (**c**) Representative images of TRAP staining showing osteoclast differentiation in Ad-Ctrl- or Ad-*Mycl*-transfected BMMs under differentiation conditions. (**d**) Quantification of osteoclasts (OCs/cm²) from cultures in 96-well plates. (**e**) ChIP analysis of Flag-Mycl binding to the *Ctsk* promoter in BMMs transfected with Ad-Ctrl or Ad-*Mycl* after 4 days of differentiation. (**f**) Luciferase reporter assay of *Ctsk* promoter activity in RAW264.7 cells transfected with Ad-Ctrl or Ad-*Mycl* for 48 h. Data are expressed as means ± SD from three independent experiments. ^*^p < 0.05, ^**^p < 0.01, ^***^p < 0.001 vs. Ad-Ctrl.

To elucidate Mycl’s role in osteoclastogenesis, we overexpressed *Mycl* in BMMs using Ad-*Mycl*. After 4 days of differentiation, qRT-PCR analysis demonstrated that *Mycl* overexpression significantly upregulated both *Mycl* and key osteoclastogenic markers (*Nfatc1*, *Ctsk*) at the mRNA level (Fig. 5a). Western blot analysis further confirmed corresponding increases in NFATc1 protein expression (Fig. 5b), suggesting that Mycl promotes osteoclast differentiation.

Functional assessment by TRAP staining revealed that *Mycl*-overexpressing BMMs generated significantly more multinucleated (≥ 3 nuclei) TRAP-positive osteoclasts compared to controls (Fig. 5c, d), indicating that Mycl overexpression promotes osteoclast differentiation.

Mechanistically, ChIP assays identified direct binding of Mycl to the *Ctsk* promoter in differentiating BMMs (Fig. 5e). This direct binding was further supported by luciferase reporter assays in RAW264.7 cells in the absence of RANKL, where Mycl overexpression significantly enhanced *Ctsk* promoter activity (Fig. 5f). The physiological relevance of this direct interaction is supported by observations from both systems: the simplified reporter assay confirms transactivation potential, while the ChIP assay in differentiating BMMs confirms its occurrence under osteoclastogenic conditions.

### Mycl overexpression counteracts GSK-mediated suppression of RANKL-induced osteoclastogenesis

**Fig. 6:**
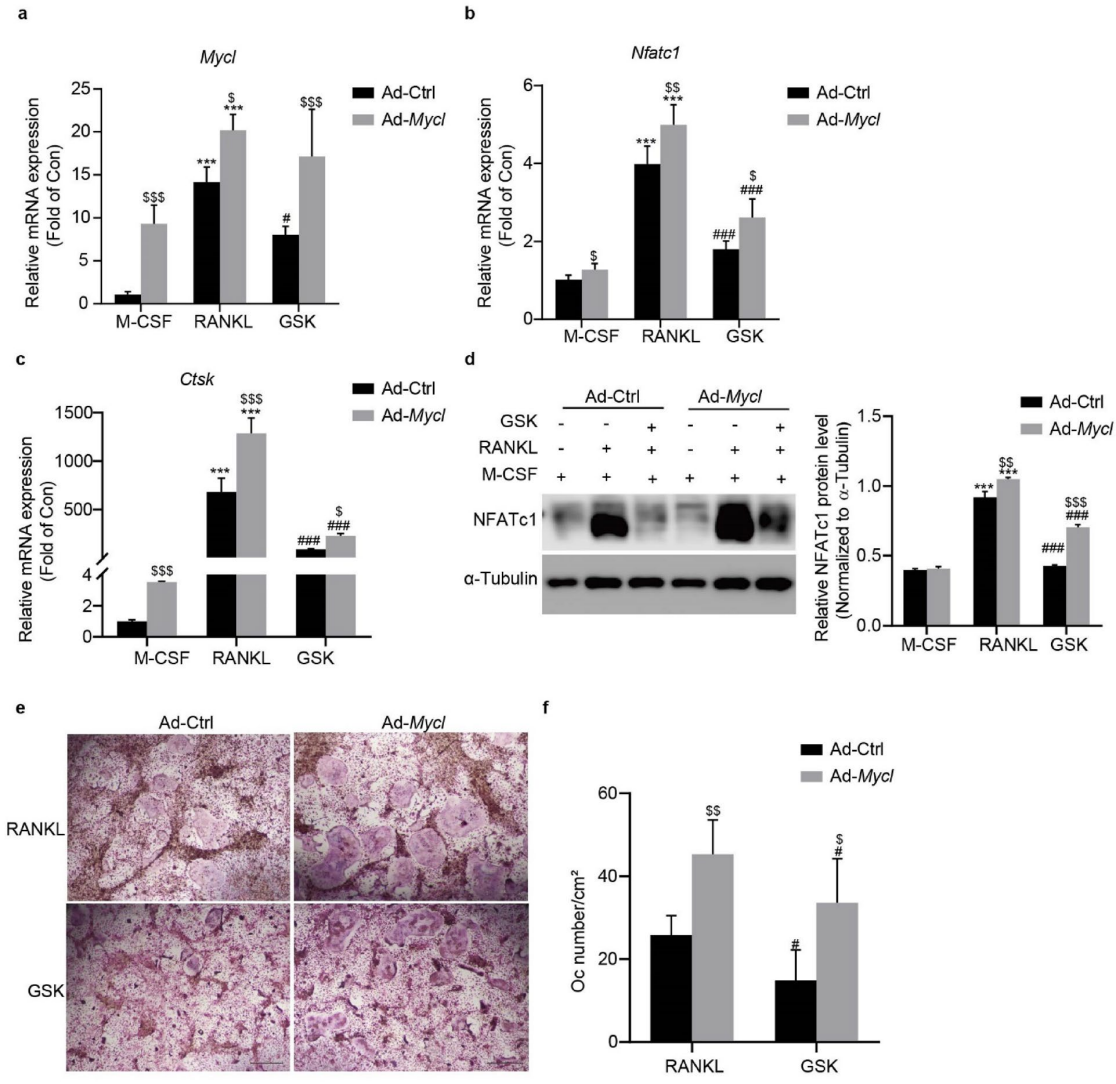
Mycl overexpression counteracts GSK-mediated suppression of RANKL-induced osteoclast-specific gene expression. (**a**–**c**) Relative mRNA levels of *Mycl* and osteoclastogenic markers (*Nfatc1*, *Ctsk*) in BMMs transfected with Ad-*Mycl* or Ad-Ctrl after 4 days of osteoclast differentiation in the presence or absence of GSK. (**d**) NFATc1 protein expression was determined by Western blot and quantified in Ad-*Mycl*- or Ad-Ctrl-transfected BMMs after 4 days of differentiation with or without GSK treatment. The blot image shows adjacent lanes from the same membrane. Band intensities were quantified with ImageJ software and normalized to α-Tubulin. Original blots are provided in Supplementary Fig. 11. (**e**) Representative images of TRAP staining showing osteoclast differentiation in Ad-*Mycl*- or Ad-Ctrl-transfected BMMs treated with or without GSK under osteoclast differentiation conditions. (**f**) Quantification of osteoclasts (OCs/cm²) from cultures in 96-well plates. Data are expressed as means ± SD for three independent experiments. ^***^p < 0.001 vs. M-CSF; ^#^*p* < 0.05, ^###^*p* < 0.001 vs. RANKL group; ^$^*p* < 0.05, ^$$^*p* < 0.01, ^$$$^*p* < 0.001 vs. Ad-Ctrl group.

To investigate whether Mycl could rescue GSK-induced inhibition of osteoclast differentiation, we performed gain-of-function experiments in RANKL-stimulated BMMs. qRT-PCR analysis demonstrated that Ad-*Mycl* not only increased *Mycl* expression but also upregulated key osteoclastogenic markers (*Nfatc1*, *Ctsk*) compared to Ad-Ctrl controls (Fig. 6a-c). Most significantly, Mycl overexpression reversed the transcriptional suppression of these genes induced by GSK treatment.

At the protein level, Western blot analysis revealed that Mycl restored NFATc1 expression in GSK-treated cells (Fig. 6d), confirming transcriptional regulation translates to functional protein changes. This rescue effect was functionally validated by TRAP staining (Fig. 6e, f). The coordinated recovery of molecular markers and cellular differentiation supports the functional importance of Mycl in sustaining osteoclastogenesis. Collectively, these results position Mycl as a key downstream effector in the Sgk1 signaling pathway, capable of rescuing osteoclastogenesis from Sgk1-inhibited suppression.

## Discussion

Building upon our pharmacological and genetic evidence, we now delineate a core signaling cascade controlling osteoclast activity. Our study describes the Sgk1-Stat3-Mycl axis as a novel regulatory pathway governing osteoclast differentiation and bone homeostasis. Through integrated pharmacological and genetic approaches, we demonstrate that Sgk1 inhibition attenuates osteoclastogenesis by disrupting Stat3-dependent Mycl expression, leading to suppressed *Ctsk* transcription and maintained bone microarchitecture. These findings position Mycl as a key molecular link between Sgk1 signaling and osteoclast function, thereby uncovering a novel regulatory mechanism in bone biology. The dual capacity to inhibit osteoclast differentiation while maintaining bone structural integrity suggests this pathway may offer therapeutic advantages over existing antiresorptive agents.

Our findings indicate that Sgk1 acts as a dual regulator of osteoclastogenesis, functioning both as: (1) a constitutive maintainer of basal differentiation (M-CSF-dependent) and (2) an amplifier of RANKL-induced activation. This is evidenced by Sgk1 inhibition reducing baseline osteoclastic gene expression while attenuating - but not abolishing - RANKL stimulation, yielding intermediate expression levels. The amplification mechanism likely involves a positive feedback loop, as RANKL itself upregulates Sgk1 expression^10^. These findings explain Sgk1’s context-dependent actions: maintaining physiological turnover during homeostasis while exacerbating pathological resorption in inflammatory states with elevated RANKL. Notably, our data reveal an osteoclast-specific therapeutic profile distinct from previous reports. While Sgk1 may promote osteoblast function in certain contexts^24,25^, our model focused on homeostatic osteoclast regulation, where PINP levels remained unchanged. This selectivity enables improved cortical bone parameters without impairing bone formation. However, the condition-specific efficacy of Sgk1 inhibition - moderate modulation of basal remodeling versus strong suppression of pathological activation - must be weighed against reported detrimental effects in inflammatory settings^11^. These differential outcomes may stem from: (1) varying RANKL/Sgk1 feedback loop activity across microenvironments, and (2) integration of additional signaling inputs in disease states. Our osteoclast-selective findings suggest Sgk1 inhibitors will be particularly suited for conditions requiring targeted anti-resorptive effects without compromising osteoblast function.

The identification of Mycl as a key downstream effector of the Sgk1-Stat3 axis refines our understanding of the functional landscape of MYC paralogs in osteoclast biology. Although c-MYC regulates osteoclastogenesis through multiple targets (e.g., TRAP, ERRα, mTORC1)^21,26,27^, our data indicate that Mycl contributes to osteoclastogenesis, at least in part through mediating Ctsk transactivation downstream of Sgk1-Stat3. The full scope of the Mycl targetome in osteoclasts remains to be mapped.

Mechanistically, our findings position Sgk1 as an upstream regulator of Stat3-Mycl signaling in osteoclasts. While Sgk1 as a serine/threonine kinase cannot directly phosphorylate Stat3 at Tyr705, our follow-up studies revealed that Sgk1 inhibition reduces Jak2 protein levels without affecting its mRNA expression (Supplementary Fig. 3a-d). As Jak2 is a key tyrosine kinase responsible for Stat3 phosphorylation, this post-transcriptional regulation of Jak2 suggests a mechanistic explanation for the observed reduction in p-Stat3 (Tyr705) upon Sgk1 inhibition. Notably, our data indicate that Src—another upstream kinase capable of phosphorylating Stat3 ^28^—is unlikely to be involved in this specific pathway, as neither total Src nor phosphorylated Src (p-Src) levels were affected by Sgk1 inhibition (Supplementary Fig. 3e). Thus, the Sgk1-Jak2 axis represents a major and distinct route to Stat3 activation in osteoclastogenesis.

Building upon Sgk1’s established role in Stat3 activation^29^, our experimental data reveal that pharmacological Sgk1 inhibition leads to reduced Stat3 phosphorylation at Tyr705, accompanied by decreased Mycl expression levels. This inhibition of Stat3 activation mechanistically explains the concomitant suppression of the established osteoclastogenic regulator Nfatc1, alongside our newly identified target Mycl. Our findings thus position the Sgk1-Stat3 axis as a key regulatory node that coordinately regulates both the canonical (Nfatc1) and novel (Mycl-Ctsk) branches of osteoclastogenic signaling. This regulatory cascade is consistent with clinical observations that Stat3 gain-of-function mutations upregulate both c-MYC and Mycl expression^18^, suggesting a broader functional relationship between Stat3 signaling and MYC family members. Our transcriptomic data reinforce this connection, showing that Sgk1 inhibition leads to coordinated downregulation of osteoclast markers (e.g., *Nfatc1*, *Ctsk*) along with reduced *Mycl* expression.

Notably, our ChIP and luciferase assays established that Mycl directly binds the *Ctsk* promoter, enhancing its transcription to sustain osteoclast differentiation. The rescue of *Nfatc1* and *Ctsk* expression upon Mycl overexpression in Sgk1-inhibited cells supports a model in which Mycl acts downstream of Sgk1-Stat3 to promote osteoclast-specific gene programs. These results support a model of hierarchical signaling (Sgk1→Stat3→Mycl→*Ctsk*) and highlight possible therapeutic targets for osteoporosis or inflammatory bone loss.

Despite delineating this novel Sgk1-Stat3-Mycl-Ctsk axis, our study has several limitations that should be acknowledged. First, our understanding of Mycl’s role relative to the canonical regulator c-Myc remains incomplete. Our data show that Sgk1 inhibition reduces Mycl expression without markedly affecting c-Myc or Mycn mRNA levels (Supplementary Fig. 4a, b). However, the persistently high c-Myc expression suggests possible compensatory mechanisms that our experimental design did not test. Furthermore, the potential for post-translational activation of c-Myc, independent of transcript abundance, remains unexplored. Thus, while Mycl contributes to osteoclastogenesis, its unique role relative to other MYC family members remains to be defined. Second, the mechanistic insights were primarily derived from murine models and *in* *vitro* cultures; future validation in human systems is essential to confirm clinical translatability. Third, while our approaches support a cell-autonomous role in osteoclasts, contributions from the bone marrow niche *in* *vivo* cannot be fully excluded. Lastly, the mechanism underlying Sgk1-mediated post-transcriptional reduction of Jak2 remains an open question.

The identification of this axis presents potential therapeutic opportunities for bone loss disorders. However, the paradoxical finding that Sgk1 inhibition exerts both anti-resorptive effects in osteoclasts (this study) and pro-resorptive effects in inflammatory contexts^14^ - where macrophage Sgk1 promotes anti-inflammatory M2 polarization via Stat3-FoxO1 signaling - highlights the complex, cell-type-specific roles of this kinase. These opposing effects suggest that successful clinical translation will require: (1) precise targeting of osteoclast-specific Sgk1 signaling pathways, (2) careful consideration of inflammatory microenvironmental factors, and (3) potential development of tissue-selective modulators that can discriminate between these dual functions. Future studies should determine whether the Stat3-Mycl node could be exploited as a downstream target to achieve osteoclast-specific inhibition while preserving Sgk1’s beneficial immunomodulatory roles.

## Supplementary Information

Below is the link to the electronic supplementary material.

Supplementary Material 1

## Data Availability

The datasets generated and analyzed during this study are available from the corresponding author on reasonable request.
