# Supplementary material for: Mycl, activated by Sgk1-phosphorylated Stat3, mediates osteoclastogenesis via Ctsk transcriptional regulation
Source: Sci Rep. 2025 Nov 20;15:40945. doi: 10.1038/s41598-025-24679-0 (PMC12635265; doi:10.1038/s41598-025-24679-0)
Supplement: Supplementary file 1 — Supplementary Material 1 [file 41598_2025_24679_MOESM1_ESM.pdf]

Supplementary Information

Supplementary Figure _1

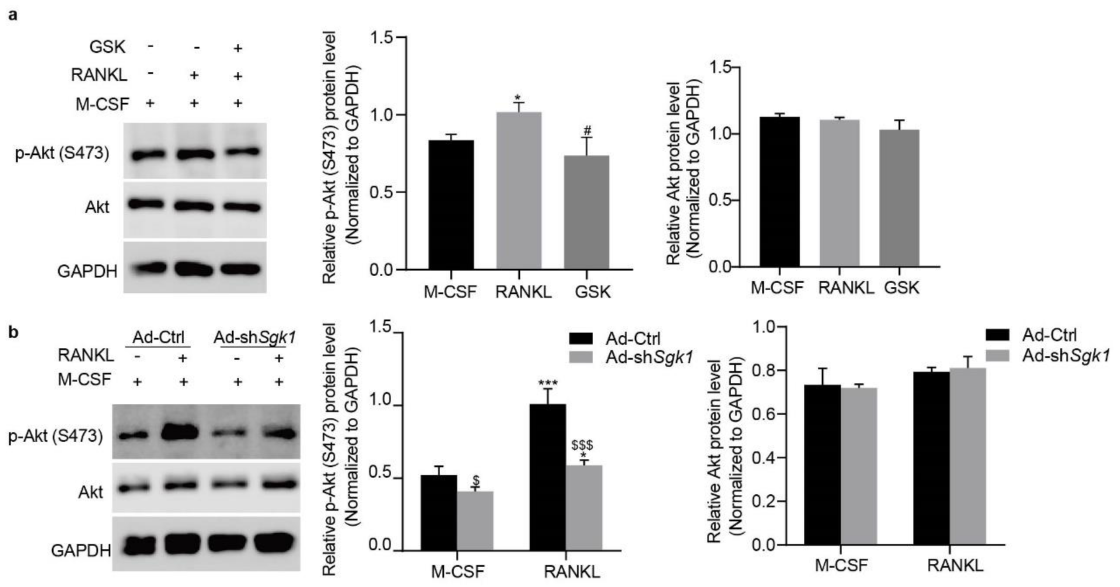

Supplementary Fig. 1. Sgk1 inhibition suppresses Akt phosphorylation in BMMs.

(a) Western blot analysis of p-Akt (S473) and total Akt protein levels in BMMs cultured under basal, differentiation, or differentiation with GSK treatment conditions for 4 days.

(b) Western blot analysis of p-Akt (S473) and total Akt protein levels in BMMs transfected with Ad-Ctrl or Ad-sh*Sgk1* and cultured under basal or differentiation conditions for 4 days.

For all Western blot panels (a, b): The blot image shows adjacent lanes from the same membrane. Band intensities were quantified with ImageJ software and normalized to the indicated loading controls (GAPDH). Original, uncropped blots are provided in Supplementary Fig. 12-13.

Data are expressed as means ± SD for three independent experiments. $^{*}p < 0.05$, $^{***}p < 0.001$ vs. M-CSF; $^{\#}p < 0.05$ vs. RANKL group; $^{\$}p < 0.05$, $^{\$\$\$}p < 0.001$ vs. Ad-Ctrl group.

Supplementary Figure _2

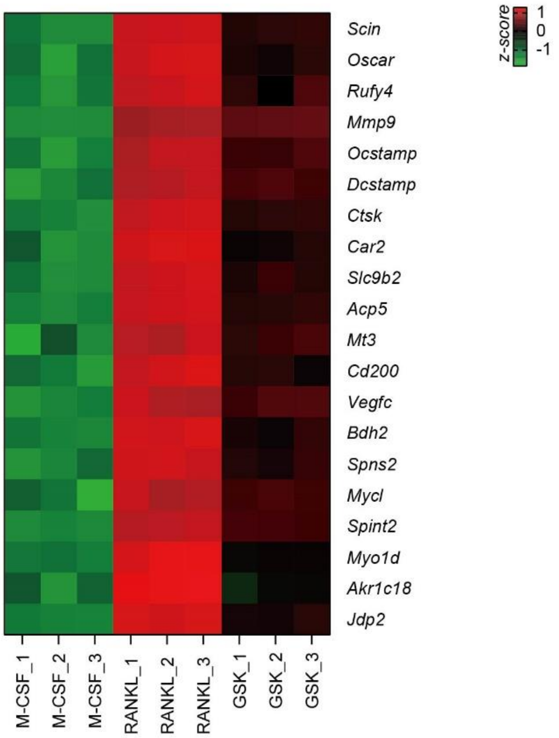

Supplementary Fig. 2. GSK reverses the core RANKL-upregulated program. Heatmap of robustly expressed (FPKM > 1 in GSK) genes that are commonly upregulated by M-CSF/RANKL and subsequently downregulated by GSK treatment, ordered by the significance of RANKL induction.

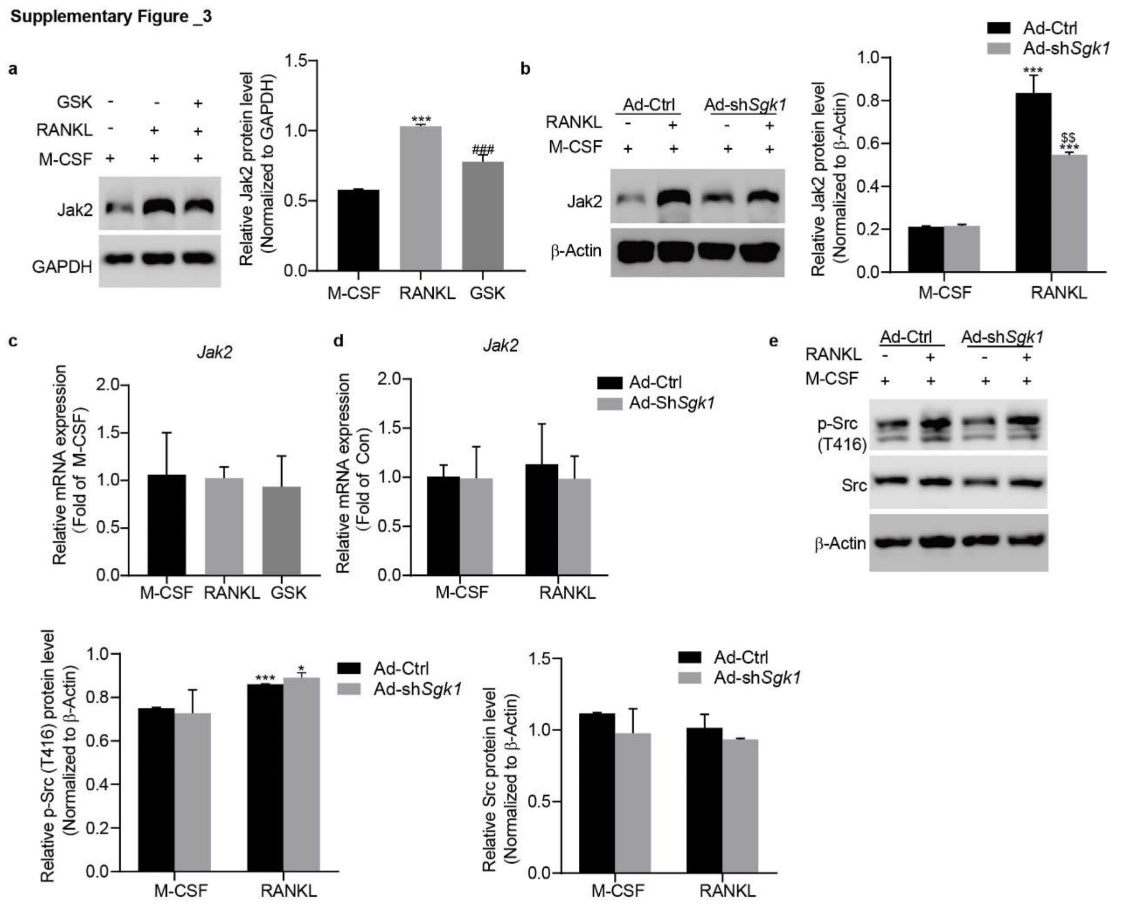

Supplementary Fig. 3. Sgk1 regulates Jak2 protein levels without affecting Src.

(a) Western blot analysis of Jak2 protein levels in BMMs under basal, differentiation, or differentiation with GSK treatment conditions for 4 days.

(b) Western blot analysis of Jak2 protein levels in BMMs transfected with Ad-Ctrl or Ad-sh*Sgk1* and cultured under basal or differentiation conditions for 4 days.

(c, d) qRT-PCR analysis of *Jak2* mRNA levels in BMMs under the indicated conditions and treatments for 4 days.

(e) Western blot analysis of p-Src (T416) and total Src protein levels in BMMs transfected with Ad-Ctrl or Ad-sh*Sgk1* and cultured under basal or differentiation conditions for 4 days.

For all Western blot panels (a, b, e): The blot image shows adjacent lanes from the same membrane. Band intensities were quantified with ImageJ software and normalized to GAPDH (panel a) or β-Actin (panels b and e) as loading controls. Original, uncropped blots are provided in Supplementary Fig. 14-16.

Data are expressed as means ± SD for three independent experiments. $^{*}p < 0.05$, $^{***}p < 0.001$ vs. M-CSF; $^{\#\#\#}p < 0.001$ vs. RANKL group; $^{\$\$}p < 0.01$ vs. Ad-Ctrl group.

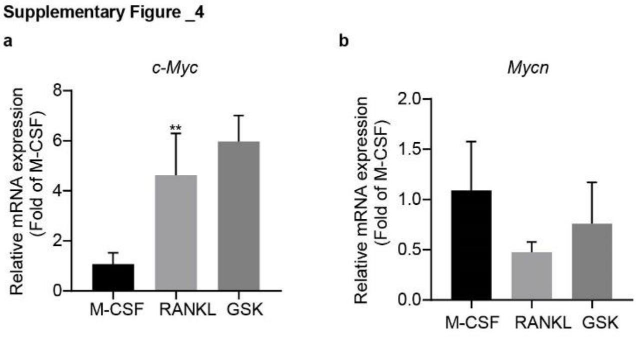

Supplementary Fig. 4. The mRNA expression of *c-Myc* and *Mycn* is unaffected by Sgk1 inhibition.

(a, b) qRT-PCR analysis of *c-Myc* (a) and *Mycn* (b) in BMMs cultured under the specified conditions for 4 days.

Data are expressed as means ± SD for three independent experiments. $^{**}p < 0.01$ vs. M-CSF.

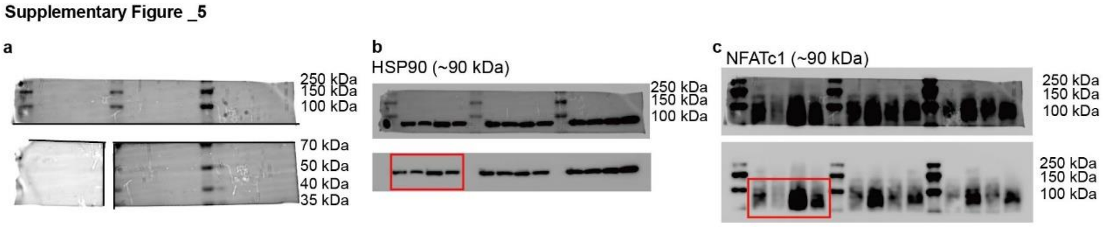

Supplementary Fig. 5. Original blots for Figure 2f.

(a) Composite image of the membrane segments after incubation, which were pre-cut by molecular weight prior to blotting (delineated by black lines).

(b, c) The specific, cropped regions corresponding to (b) HSP90 and (c) NFATc1 that were used for the final presentation in Figure 2f. The red boxes indicate the exact areas presented. The membrane shown in (c) was stripped and re-probed for NFATc1 following the HSP90 detection in (b). Molecular weight markers (kDa) are indicated.

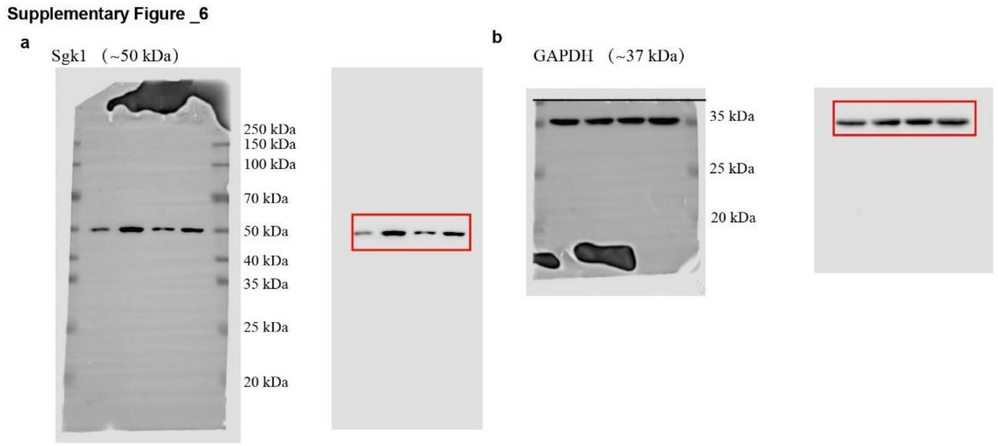

Supplementary Fig. 6. Original blots for Figure 3b.
(a) The membrane was first probed for Sgk1.
(b) The same membrane was subsequently segmented (delineated by black lines) and the lower portion was probed for GAPDH as a loading control.
Red boxes indicate the specific regions presented in Figure 3b. Molecular weight markers (kDa) are indicated.

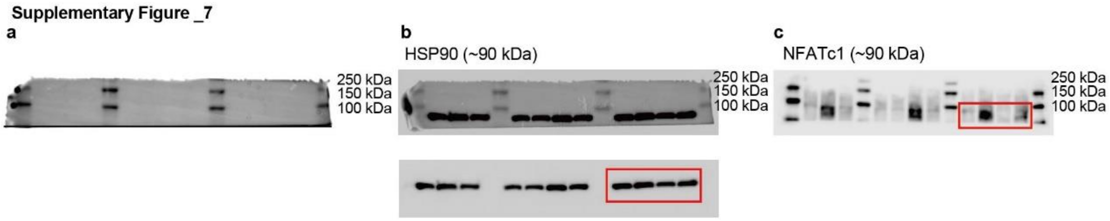

Supplementary Fig. 7. Original blots for Figure 3h.
(a) The membrane was segmented by molecular weight prior to blotting, as indicated by the black lines. The upper segment (approximately 70-250 kDa) was used for subsequent analysis.
(b, c) The upper membrane segment was sequentially probed for (b) HSP90 and, after stripping, for (c) NFATc1.
Red boxes indicate the specific regions presented in Figure 3h. Molecular weight markers (kDa) are indicated.

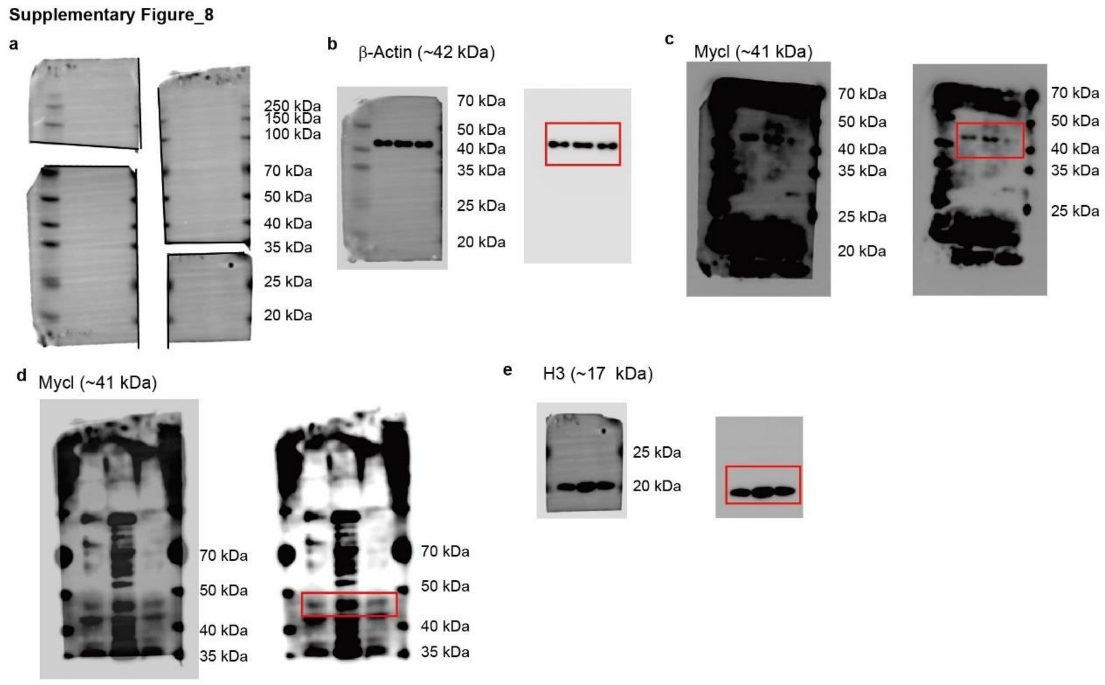

Supplementary Fig. 8. Original blots for Figure 4f.

(a) Composite image of the original membrane, which was segmented vertically after transfer to facilitate separate probing of cytoplasmic and nuclear fractions. Cut boundaries are indicated by black lines.

(b-e) The resulting membrane segments were probed as follows: (b) Cytoplasmic fraction for β-Actin; (c) The same cytoplasmic membrane after stripping and reprobing for Mycl; (d) Nuclear fraction (upper segment) for Mycl; (e) Nuclear fraction (lower segment) for Histone H3.

Red boxes indicate the specific regions presented in Figure 4f. Molecular weight markers (kDa) are indicated.

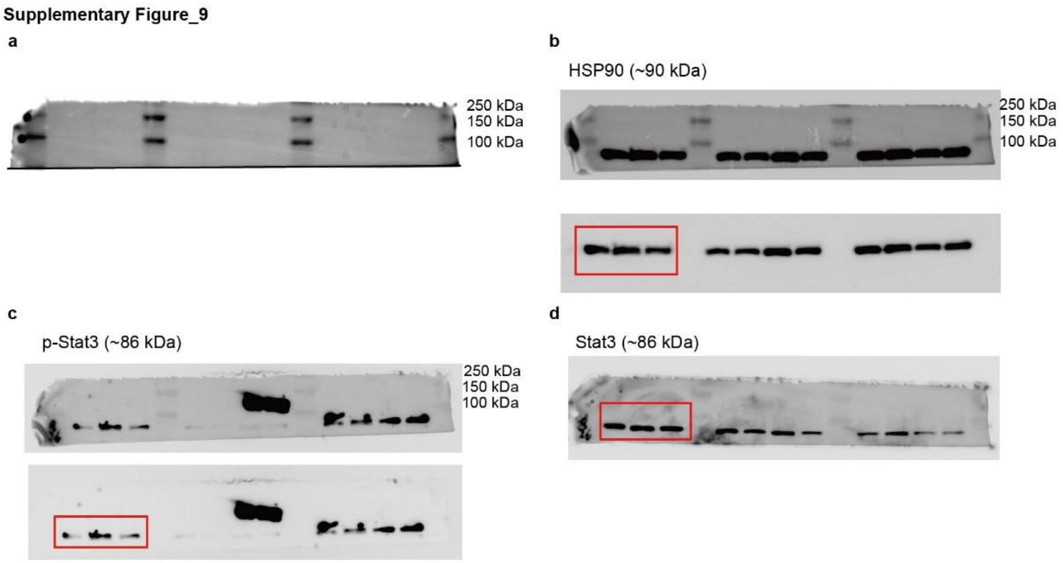

Supplementary Fig. 9. Original blots for Figure 4l.

(a) The upper segment (approximately 70-250 kDa) of the membrane, which was segmented by molecular weight prior to blotting. Cut boundaries are indicated by black lines.

(b-d) This membrane segment was sequentially probed for (b) HSP90, (c) p-Stat3 (Tyr705) after stripping, and (d) total Stat3 after a final stripping procedure.

Red boxes indicate the specific regions presented in Figure 4l. Molecular weight markers (kDa) are indicated.

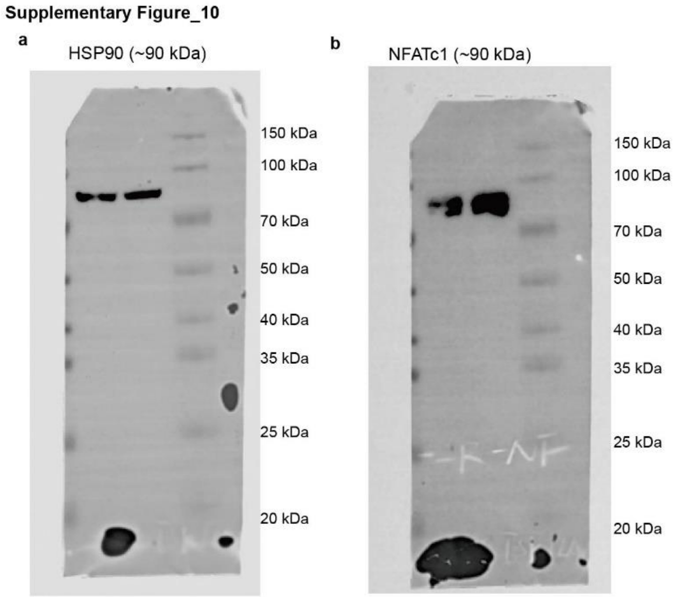

Supplementary Fig. 10. Original blots for Figure 5b.

(a, b) Western blot analysis of the indicated proteins in BMMs overexpressing Mycl. The membrane was sequentially probed for (a) HSP90 and, after stripping, for (b) NFATc1.

Red boxes indicate the specific regions presented in Figure 5b. Molecular weight markers (kDa) are indicated.

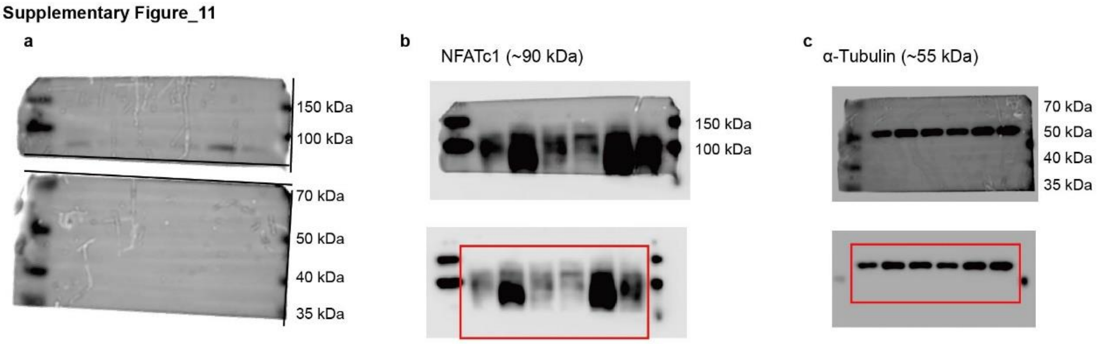

Supplementary Fig. 11. Original blots for Figure 6d.

(a) The membrane was segmented after transfer (delineated by black lines) to allow for simultaneous probing of different targets.

(b, c) The resulting segments were probed for (b) NFATc1 and (c) α-Tubulin.

Red boxes indicate the specific regions presented in Figure 6d. Molecular weight markers (kDa) are indicated.

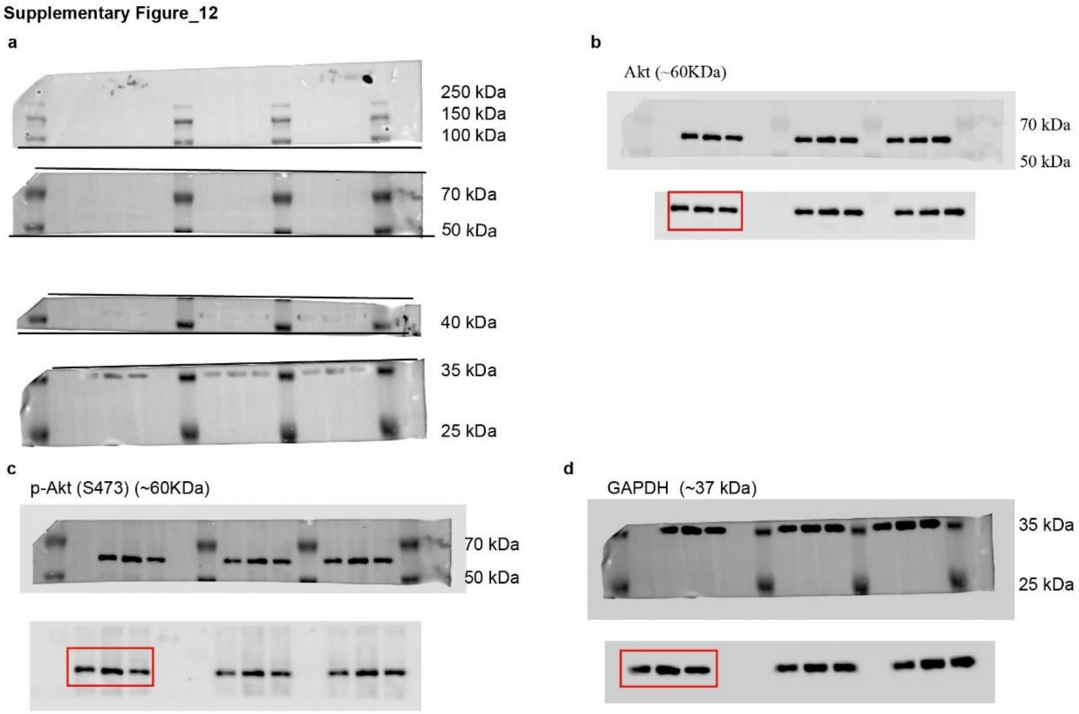

Supplementary Fig. 12. Original blots for Supplementary Fig. 1a.

(a) The membrane was segmented by molecular weight prior to blotting (delineated by black lines) to facilitate separate antibody incubation.

(b-d) The membrane segments were probed as follows: (b) Middle-upper segment for total Akt; (c) The same segment as in (b) after stripping and reprobing for p-Akt (S473); (d) Lower segment for GAPDH.

Red boxes indicate the specific regions presented in Supplementary Fig. 1a.

Molecular weight markers (kDa) are indicated.

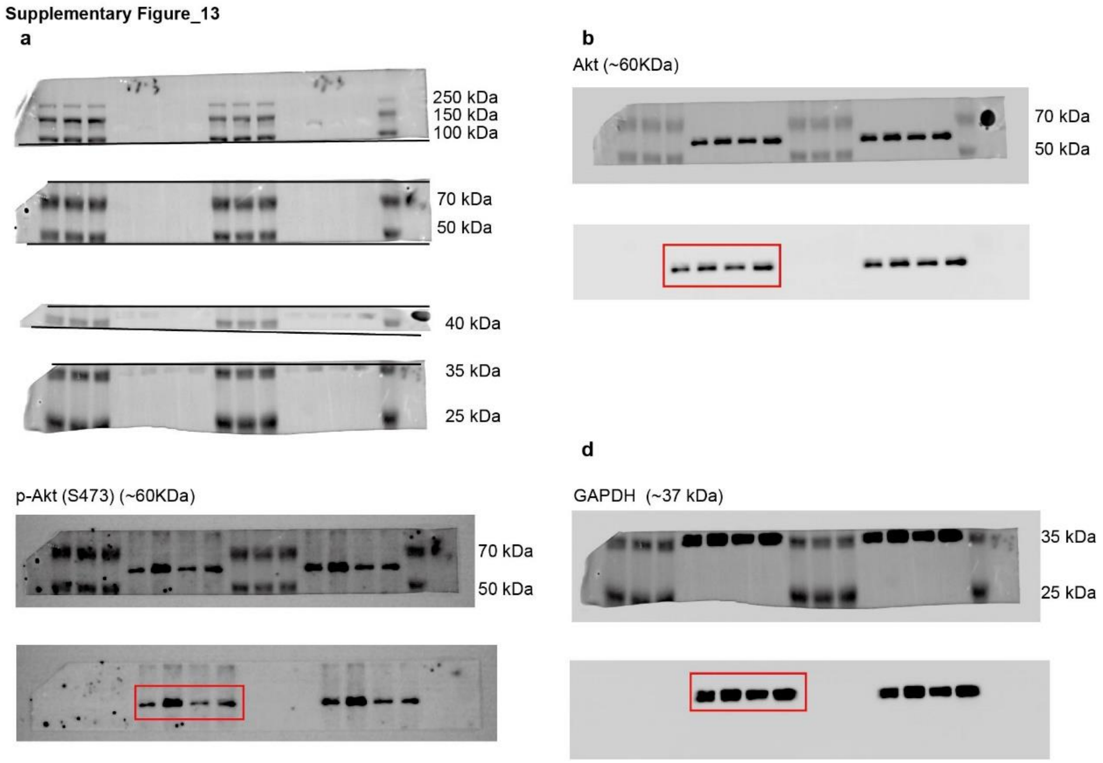

Supplementary Fig. 13. Original blots for Supplementary Fig. 1b.

(a) The membrane was segmented by molecular weight prior to blotting (delineated by black lines).

(b-d) The membrane segments were probed as follows: (b) The 50-70 kDa segment for total Akt; (c) The same segment as in (b) after stripping and reprobing for p-Akt (S473); (d) The 25-35 kDa segment for GAPDH.

Red boxes indicate the specific regions presented in Supplementary Fig. 1b.

Molecular weight markers (kDa) are indicated.

Supplementary Figure_14

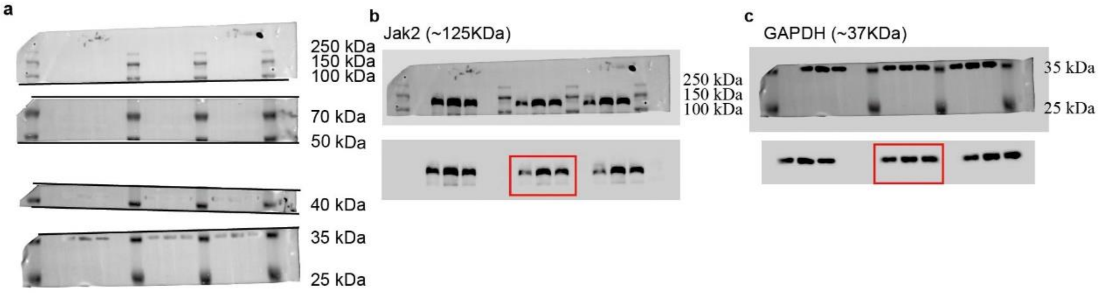

Supplementary Fig. 14. Original blots for Supplementary Fig. 3a.

(a) The membrane was segmented by molecular weight prior to blotting (delineated by black lines).

(b, c) The membrane segments were probed for (b) Jak2 and (c) GAPDH.

Red boxes indicate the specific regions presented in Supplementary Fig. 3a.

Molecular weight markers (kDa) are indicated.

Supplementary Figure_15

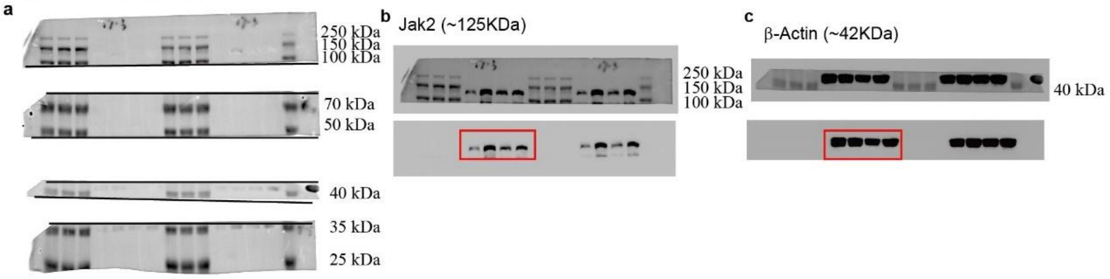

Supplementary Fig. 15. Original blots for Supplementary Fig. 3b.

(a) The membrane was segmented by molecular weight prior to blotting (delineated by black lines).

(b, c) The membrane segments were probed for (b) Jak2 and (c) β-Actin.

Red boxes indicate the specific regions presented in Supplementary Fig. 3b.

Molecular weight markers (kDa) are indicated.

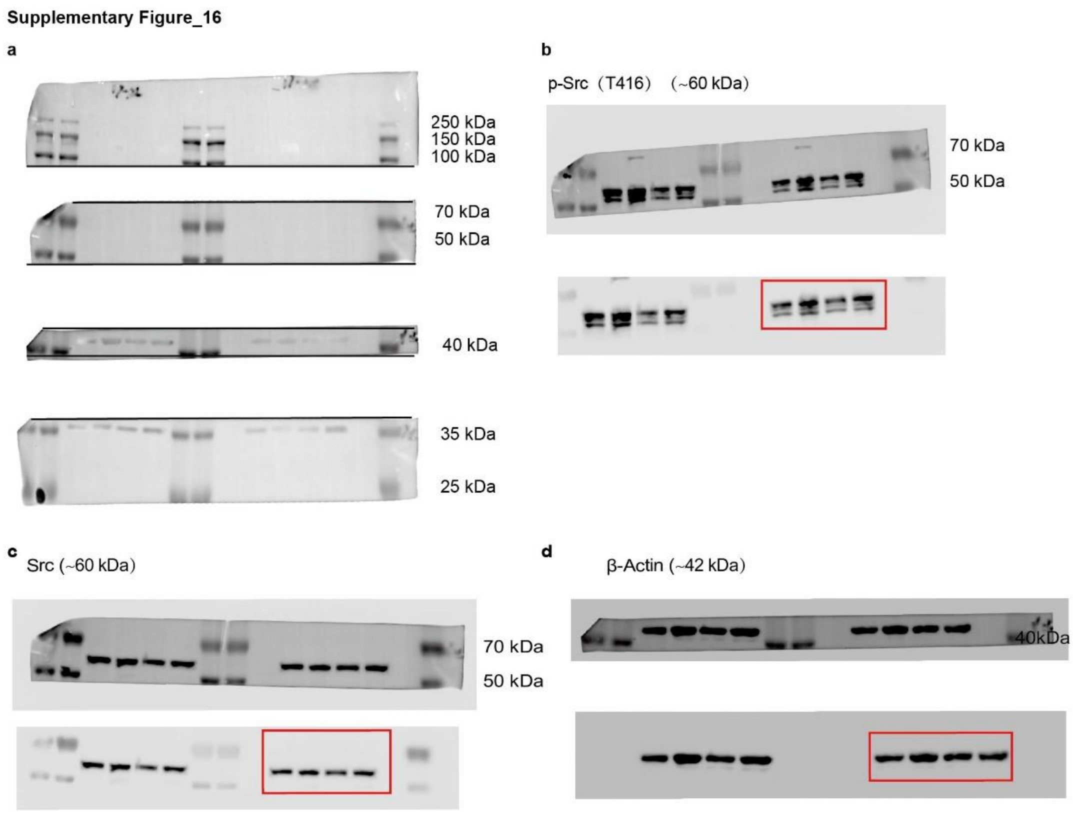

Supplementary Fig. 16. Original blots for Supplementary Fig. 3e.

(a) The membrane was segmented by molecular weight prior to blotting (delineated by black lines).

(b-d) The membrane segments were probed as follows: (b) The 50-70 kDa segment for p-Src (T416); (c) The same segment as in (b) after stripping and reprobing for total Src; (d) The 40-50 kDa segment for β-Actin.

Red boxes indicate the specific regions presented in Supplementary Fig. 3e.

Molecular weight markers (kDa) are indicated.
